# Systematic Review of Contemporary Theories Used for Co-creation, Co-design and Co-production in Public Health

**DOI:** 10.1093/pubmed/fdad046

**Published:** 2023-05-05

**Authors:** Katrina Messiha, Mai J M Chinapaw, Hans C F F Ket, Qingfan An, Vinayak Anand-Kumar, Giuliana R Longworth, Sebastien Chastin, Teatske M Altenburg

**Affiliations:** Department of Public and Occupational Health, Amsterdam Public Health Research Institute, Amsterdam UMC, Vrije Universiteit Amsterdam, Amsterdam, The Netherlands; Department of Public and Occupational Health, Amsterdam Public Health Research Institute, Amsterdam UMC, Vrije Universiteit Amsterdam, Amsterdam, The Netherlands; Medical Library, Vrije Universiteit Amsterdam, Amsterdam, The Netherlands; Department of Community Medicine and Rehabilitation, Umeå University, Umeå, Sweden; Department of Psychology and Methods, Jacobs University Bremen, Bremen, Germany; Faculty of Psychology, Education and Sport Sciences Blanquerna, Universitat Ramon Llull, Barcelona, Spain; School of Health and Life Sciences, Glasgow Caledonian University, Glasgow, UK; Department of Movement and Sports Science, Ghent University, Ghent, Belgium; Department of Public and Occupational Health, Amsterdam Public Health Research Institute, Amsterdam UMC, Vrije Universiteit Amsterdam, Amsterdam, The Netherlands

**Keywords:** co-creation, co-design, co-production, public health, theoretical framework, theory

## Abstract

**Background:**

There is a need to systematically identify and summarize the contemporary theories and theoretical frameworks used for co-creation, co-design and co-production in public health research.

**Methods:**

The reporting of this systematic review follows the Preferred Reporting Items for Systematic Reviews and Meta-Analyses. Given substantial interest in and application of co-creation, co-design and co-production, we searched PubMed, CINAHL, Scopus and APA PsycINFO from 2012 to March–April 2022. A quality assessment and data extraction for theory content was performed.

**Results:**

Of the 3763 unique references identified through the comprehensive search strategy, 10 articles were included in the review: four articles named co-creation, two articles named co-creation and co-design, two articles named co-production and co-design, and two articles named co-design. Empowerment Theory was employed by two articles, whereas other theories (*n* = 5) or frameworks (*n* = 3) were employed by one article each. For the quality assessment, eight articles received a strong rating and two articles received a moderate rating.

**Conclusion:**

There is little indication of theory applications for the approaches of co-creation, co-design and co-production in public health since 2012, given 10 articles were included in this review. Yet, the theories described in these 10 articles can be useful for developing such co-approaches in future public health research.

## Background

Public health is aimed at preventing disease whilst prolonging life and promoting health in society.^1^ Co-creation, co-design and co-production have much potential for use in public health research, as claimed to more actively involve stakeholders and their perspectives in the context of proposed initiatives.^2–4^ Existing evidence maintains that such co-approaches can offer benefits to research processes and outcomes, as well as involved stakeholders.^2–4^ For instance, it can lead to more relevant knowledge production and better tailored solutions. Although the terms are overly used^5^ and interchangeable, they refer to different approaches.^6–8^ Hence, this review nominates Vargas *et al*.’s^7^ definitions.

Co-creation is defined as an all-encompassing principle about collaboration and innovative problem-solving among various stakeholders across all initiative phases (e.g., from problem identification to evaluation). Co-design is distinguished as the active collaboration among stakeholders relating to solution design, given a pre-determined problem, whilst co-production is about engaging stakeholders in the implementation of previously set solutions to an already agreed problem, in prioritizing the optimal usage of available resources. In addition, crucial differences exist in terms of stakeholder roles and the nature of engagement in such co-approaches.^7^^,^^9^^,^^10^ Yet, scholars have lacked in systematically and effectively contributing to theory-building of co-creation.^11–13^ This may be partly due to inconsistencies and confusion in how these terms are used, coupled with potential knowledge fragmentation between fields, given the interdisciplinary nature of co-creation.

To advance co-creation, it is crucial to build on (explicit) theory as to enable the realization and accumulation of scientific knowledge, which can lead to evidence about best practices as well as a shared understanding of the co-creation approach.^14–16^ Theory can offer more transparency, consistency and rigour in the research process, for example, helping to evaluate impact on co-creators and of the co-created initiatives. Whilst the most extensively cited guidance posits that public health intervention development must be underpinned by theory and an evidence base,^3^ understanding the different theories about the application of co-creation approaches,^17^^,^^18^ across all initiative phases, is key. To our knowledge, there is no systematic review consolidating the contemporary theories used for co-creation, co-design and co-production (in public health research) to date.

### Aim

We aim to identify and summarize the contemporary theories/ theoretical frameworks used for co-creation, co-design and co-production in public health research. We used the definitions of ‘theory’ and ‘theoretical framework’ from Kivunja^19^ for guiding this review.

- Theory covers connected concepts, definitions and hypotheses to depict a systemized understanding of a given phenomenon via the connection to variables, with the aim of both delineating and predicting such phenomena.
- Theoretical frameworks provide a structure that encapsulates concepts and theories, which results from historically tested and disseminated knowledge.

## Methods

The reporting of this systematic review aligns with the Preferred Reporting Items for Systematic Reviews and Meta-Analyses (PRISMA).^20^ This review progressed on the basis of a registered protocol (PROSPERO ID: CRD42022324074).^21^

### Search strategy

A comprehensive search (Appendix 1) was performed by lead author (K.M.) and a medical information specialist (H.K.) in four databases: PubMed, Ebsco/ CINAHL, Ebsco/ APA PsycINFO and Elsevier/ Scopus, from 2012 to March–April 2022 given the recent upsurge in general interest and application of these approaches in various fields over the last 10 years.^22–24^ The search included controlled and free-text terms for synonyms of ‘theory’ and ‘co-creation’ or ‘co-design’ or ‘co-production’, with no restriction for methodology or language. The reference lists of the included articles from the full-text screening were reviewed to identify additional relevant articles.

### Selection of articles

Original studies (including protocols) had to fulfil the following criteria: contain an explicit reference to named theory/ theoretical frameworks used for co-creation, co-design and co-production in the public health research. This includes theory use for data analysis/ interpretation relating to comprehending feedback on co-creation, co-design and co-production from the participating stakeholders. Using this review’s working definition of public health, the publications were to explicitly use the terms ‘co-creation’, ‘co-design’ or ‘co-production’, where theory/ theoretical frameworks were to relate to any or all parts of its approach or its particular (direct) elements.

Given theory/ theoretical frameworks about the content of the intervention or product, for example, behaviour change theories (The Capability, Opportunity, Motivation, Behaviour (COM-B) model; Behavioural Change Wheel, etc.) were excluded, in addition to meta-theories. Grounded Theory, Theory of Change and its synonyms, such as programme theory, were also excluded as rendered a methodology, not a theory.^25^^,^^26^

## Screening and study selection

### Electronic database and reference checking

Duplicate articles were excluded by a medical information specialist (H.K.) using Endnote X20.0.1 (Clarivate), following the Amsterdam Efficient Deduplication method^27^ and the Bramer method.^28^ All references were then uploaded to Rayyan systematic software,^29^ to perform screening.

The lead author (K.M.) screened the titles and abstracts of all articles against the criteria. Duplicate screening was performed by one of three co-authors (Q.A., G.L., V.A.K.), each screening one-third of all articles. Next, the full-text screening was similarly conducted using all articles remaining—with the lead author (K.M.) having screened all full-text articles and three co-authors (Q.A., V.A.K., S.C.) performed double screening. Any differences in screening assessment were solved through deliberation among the reviewers. No additional references were identified from scanning the reference list of the included articles, as screened by lead author (K.M.) and a co-author (G.L.).

### Quality assessment

Two authors (K.M. and M.C. or T.A.) rated the methodological quality of the included studies using the quality assessment tool proposed by Bergeron *et al*.^30^ K.M., M.C. and T.A. pilot-tested the methodologic quality assessment to ensure the following items was interpreted consistently:

(i) Is the methodology identified and justified?
(ii) Was a theoretical lens or perspective used to guide the study, with a reference provided?
(iii) Is the theory/ theoretical framework clearly described?
(iv) Is the theory/ theoretical framework easily linked to the problem (i.e. co-creation, co-design or co-production)?
(v) If an explicit theoretical framework is used, are the concepts adequately defined?
(vi) Are the relationships among the concepts clearly operationalized for the context under study?

These questions necessitate a scoring of ‘yes’, ‘somewhat’ (i.e. partial information) or ‘no’. The ‘yes’ responses to the questions were added up as a final score. An overall score of three or less in the ‘yes’ responses was categorized as ‘moderate’, and an overall score of 4–6 was categorized as ‘strong’. The lead author (K.M.) assessed 100% of the included articles, and two co-authors (M.C., T.A.) each assessed 50% of the articles.

### Data extraction: theory analysis

Theory analysis took a modified approach from Nadalin Penno *et al*.’s work^31^—previously adapted from Walker and Avant.^32^ The theory analysis was 100% performed by the lead author (K.M.), alongside two co-authors (M.C., T.A.) who each extracted from 50% of the articles. K.M., M.C. and T.A. piloted-tested the theory analysis to ensure the following items were interpreted consistently:

(i) Publication year
(ii) Country of origin
(iii) Whether article pertains to co-creation/ co-design/ co-production
(iv) Name of the explicit theory/ theoretical framework, including any citation reference
(v) Rationale for the use of the theory/ theoretical framework
(vi) Delineation of how the theory/ theoretical framework is used
(vii) Methodological approach and
(viii) Study setting/ context

### Data analysis

The modified theory content fitting the eight sections of the extraction table of the included articles were presented in summary descriptions. Named theories were categorized based on whether the included article explicitly referenced co-creation/ co-design/ co-production.

## Results

**Fig. 1:**
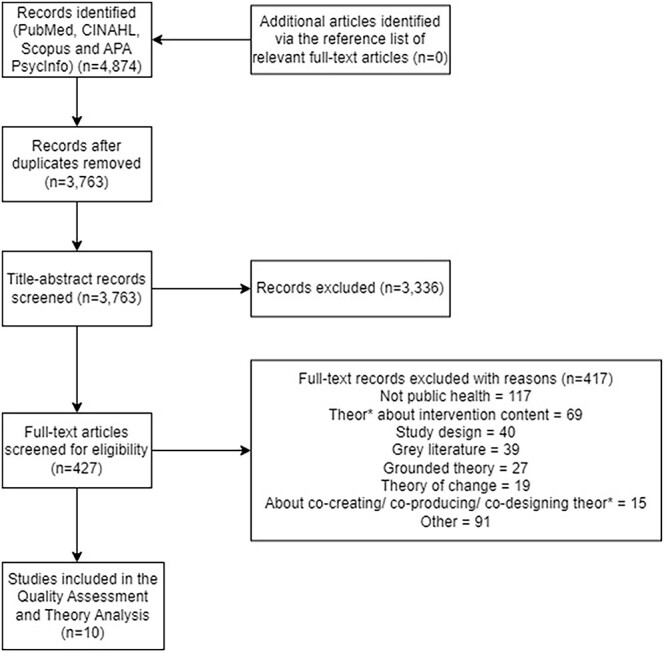
PRISMA flow diagram.

Figure 1 shows the PRISMA flow diagram presenting the screening process.

The search strategy identified 427 articles for the full-text review phase. Ten articles fulfilled the inclusion criteria, hence included in this review.

### Quality assessment

Eight articles were rated strong and two articles received a moderate rating (Appendix 2). Reasons for a moderate score include not justifying the choice of co-creation/ co-design/ co-production approach, the theory not easily being linked to co-creation/ co-design/ co-production, the concepts not being adequately defined about the use of explicit theoretical framework and the relationships among concepts were not clearly operationalized for the study context.

### Summary of studies included

Table 1 displays the findings based on the Theory Content Analysis of included articles. Regarding the country of origin, one article was based in the Netherlands,^33^ two were based in both the Netherlands and one other country, Turkey^47^ and Hungary,^37^ two were based in Denmark,^43^^,^^45^ one originated from the USA,^35^ two from UK,^50^^,^^53^ one from Scotland (Glasgow) and Australia,^55^ and one from Canada.^57^

**Table 1:** Theory Content Analysis of included articles, sorted by referenced explicit term (co-creation/ co-design/ co-production), name of the theory/ theoretical framework and study reference

Study reference (author and publication year) + Country of origin (publication)	Does the article pertain to co-creation, co-design or co-production? Specify which	Name of the explicit theory/ theoretical framework including any citation reference	Rationale for use of the theory/ theoretical framework (give summary)	Delineation of how the theory/ theoretical framework is used (give summary)	Methodological approach/ study design used	Study setting/ context used (public health context/ setting/ target population, etc.)
Anselma *et al*.^33^ The Netherlands	Co-creation	Empowerment Theory^34^	Relates to the RE-AIM framework the article uses—specifically the dimension of empowerment. Also to comprehend the influence of Youth-led Participatory Action Research (YPAR) on children’s empowerment	Empowerment theory used for focus groups (e.g., protocol development) and interview guide and as a means of evaluating the study on constructs like empowerment	Process evaluation (qualitative) of YPAR	School and community settings
Cueva *et al*.^35^ USA	Co-creation	Empowerment Theory^36^	This theory was employed in order to offer culturally respectful education for empowering individuals and promoting wellness activities. Further to this, the theory helps to promote wellness with, and for, Alaskans by empowering Alaska’s Community Health Aides/ Practitioners (CHA/ Ps) and inspiring positive behavioural change among CHA/ Ps, their patients and their communities, to both reduce cancer risk and support those who face cancer	The surveys used in this study were developed/ informed by empowerment theory, for instance: ‘designed to involve participants as professional collaborators from these first steps in articulating what a culturally responsive distance-delivered cancer education course may look and feel like’ (427)	Survey research (qualitative) design	Culturally sensitive intervention (education course about cancer and care) development
Koops van’t Jagt *et al*.^37^ Hungary and The Netherlands	Co-creation	Social Learning Theory^38–40^ and Narrative Theory^40–42^	The theory is said to be compatible with the method (photo stories): e.g., ‘through social interaction with peers, family members, or their doctors, the main characters came up with solutions and communication strategies to deal with these barriers. In this way, our photo stories were in line with theories on social learning’ (76). In addition, the article asserts that narrative communication employs story structures that offer a familiar mode of interaction. Thus, a narrative-based intervention could be easier to process	Photo stories, the method, which was employed for co-creation with the target group, was underpinned by social learning theory. Narrative theory was utilized in the planning and production research phase where narrative elements important for the theory were discussed	Qualitative methodology	Older adults and communication intervention development; patient communications in general practice
Handberg *et al*.^43^ Denmark	Co-creation	Symbolic interactionism as the theoretical framework^44^	Authors argue that this theoretical framework was a powerful lens regarding epistemological integrity for allowing access into knowledge looked for and expanding the choices for analysis and data interpretation	Used alongside the interpretive description methodology, symbolic interactionism as the theoretical framework was adopted during the field work, in developing the observation guide and throughout the analyses	Qualitative ethnographic fieldwork using interpretive description	Community-based rehabilitation
Hoeeg *et al*.^45^ Denmark	Co-creation and co-design	‘Social Effectiveness of Interventions’^46^	For understanding social effectiveness of interventions, and thereby social relations—their embeddedness and interference. Helps to highlight dynamics and social processes	Theory used as an analytical method for understanding the co-creation process and investigating merits and limitations of the Design-based Research (DBR) methodology	Process evaluation (qualitative)	Community of pre-school children
Garton *et al*.^47^ The Netherlands and Turkey	Co-creation (and co-design for the research questions)	Realist evaluation (theoretical) framework^48^^,^^49^	Justified for accounting for strategic changes/ fluidity over time in the intervention implementation. Also, it enables sense-making between context, mechanisms and outcome	The context–mechanism–outcome framework of the realist evaluation was superimposed on the traditional logical framework (log frame) tool used for programme evaluation to create the conceptual framework for this study	Mixed-methods case study design	NCD (non-communicable disease) prevention in Istanbul (Turkey)
Duke *et al*.^50^ UK	Co-production and co-design	Normalization Process Theory^51^^,^^52^ (integrated with Participatory Learning and Action (PLA) Research approach)	The theory was combined with the Participatory Learning and Action (PLA) Research approach, which was justified since the authors articulate that this blended approach can positively influence the intervention design quality and implementation, by allowing for diverse knowledge and expertise sources are incorporated	In the pilot implementation (PLA cycle) phase, the data collection was informed by the theory	Participatory Learning and Action Research	End-of-life care in the UK
Clarke *et al*.^53^ UK	Co-production and co-design (but predominantly co-production)	Interactional ritual change theory^54^	Complements existing research on the significance of ensuring stakeholder inclusivity, by underscoring the notion of everyday routines/ rituals in facilitating a sense of belonging. In addition, it provides a unique perspective relating to situated co-production practices	The theory was used in an informative capacity in order to recognize the two types of ‘interlinked inclusivity’, that is, relational and emotional. The theory is used as part of the data analysis for investigating how co-production takes place via routine and ritualistic patterns of everyday practices, with a view to enable sustainable and inclusive initiatives for research	Ethnographic comparative case study approach	Co-production related to health research projects (*n* = 4) in various settings
Farmer *et al*.^55^ Scotland and Australia	Co-design	Social Innovation Theory^56^	The theory is justified for use in relation to its ability to underscore that comprehending initiatives co-designed with communities as social innovation could aid in recognizing useful initiatives in the co-design processes to support change in public health. Further to this, to examine how instrumental ‘top-down’ community participation processes (driven by regional commissioners) enable diverse grassroots participants to gather, learn from each other, share knowledge and adapt ideas to new contexts and to consider what happens to innovations once they are planned and enacted	This theory was employed for analysis in order to explore co-design cases at three phases (innovation growth, development and sustainability/ diffusion). Specifically, it is used to investigate and comprehend what happened at each phase of developing the innovation. The authors created a model of the grassroots social innovation process in which they contain three key phases and elements	Co-design process with community members, including co-design workshops, implementation group meetings and cross-community meetings	Community setting (6 communities) in regard to health service development
Latulippe *et al*.^57^ Canada	Co-design	Amartya Sen’s theoretical framework of social justice^58^	The theoretical framework of social justice given by Amartya Sen was used given its informative for co-design potential, with respect to the democratic process and as a conversion factor. Further to this, including future users (FUs) in digital tool development helps to consider the interests and capacities of FUs and their attitudes, beliefs, values and expectations. This increases the probability of forming a tool which is universally accepted	This theoretical framework was used as a means of guiding the aims in investigating how co-design can assist an inclusive intervention (eHealth tool) for caregivers of older persons rendered functionally dependent	Participant observation study with co-design sessions/ participatory meetings	Community setting and contextualized to the development of eHealth tools for caregivers (of functionally dependent older people)

Four articles referred to co-creation.^33^^,^^35^^,^^37^^,^^43^ Two articles referred to both co-creation and co-design.^45^^,^^47^ Two articles referred to both co-production and co-design.^50^^,^^53^ Two articles referred to co-design.^55^^,^^57^

All included studies used a predominantly qualitative study design, in terms of process evaluation of youth participatory research,^33^ design-based research,^45^ mixed-methods case study design incorporating mainly quantitative methods with a qualitative assessment,^47^ survey design,^35^ qualitative methodology,^37^ participatory learning and action research,^50^ ethnographic approach^43^^,^^53^ and co-design processes^55^^,^^57^ (e.g., workshops).

The studies demonstrate various collaborative approaches across the school and community setting,^33^ community of pre-school children,^45^ NCD (non-communicable disease) prevention,^47^ culturally sensitive intervention development,^35^ older adults involved in communication intervention,^37^ community-based rehabilitation,^43^ end-of-life care,^50^ community setting in health service development,^55^ health research projects in different settings,^53^ community setting regarding caregivers and eHealth tool development.^57^

Table 2 presents the summaries of the named theory/ theoretical framework.

**Table 2:** Theory summaries given by the included articles

Study reference	Theory summaries
Anselma *et al*.^33^	**Empowerment Theory** ^34^ is about people’s ability to gain comprehension and control over personal, social, economic and political aspects in order act towards improving their life.
Cueva *et al*.^35^	**Empowerment Theory** ^36^ relates to enhancing wellness and bettering problems, offering opportunities for participants to developing their knowledge and skills, as well as engage professionals as collaborators as opposed to authoritative experts.
Koops van’t Jagt *et al*.^37^	**Social Learning Theory** ^38–40^ regards the notion that observation and modelling mainly influence how and why individuals learn. **Narrative Theory**^40–42^ refers to using story-telling as a structure for a familiar way of interacting, which may lead to increased personal involvement and give users role-models and phase-by-phase scenarios. Thus, it is related to acquiring learning through experiences.
Handberg *et al*.^43^	**The Theoretical Framework of Symbolic Interactionism** ^44^ posits that human beings act towards things from personal meaning in life, and that this meaning arises from interactions with others, where such meanings are adapted from an interpretative process used by the encountering individual.
Hoeeg *et al*.^45^	**‘Social Effectiveness of Interventions’ Theory** ^46^ relates to an intervention only being socially effective if it generates a ‘shared understanding between researchers and professionals and reconfigures the social relationships between researchers and professionals through processes of “exchange”’ (2).
Garton *et al*.^47^	**Realist Evaluation (Theoretical) Framework** ^48^ ^,^ ^49^ concerns the assessment of real-life interventions in a different way from traditional scientific experiments. A realist evaluation asks, ‘what works for whom, under what circumstances, and how?’ and answers these questions through a process of ‘sense-making’ between the context, mechanisms and outcome patterns of a programme.
Duke *et al*.^50^	**Normalization Process Theory** ^51^ ^,^ ^52^ is a structural approach to comprehending the factors that both serve and restrict implementation. It has constructs around coherence, cognitive participation, collective action and reflexive monitoring.
Clarke *et al*.^53^	**Interactional Ritual Change Theory** ^54^ relates to the way ‘symbolic meanings, beliefs and norms are transmitted and reinforced through relative stable patterns of social interaction or routine’ (236).
Farmer *et al*.^55^	**Social Innovation Theory** ^56^ is relayed ‘as involving collaborations to co-design and implement solutions to social problems, particularly at local level’ (2). Relevantly, co-produced solutions are reinforced to have positive societal effects (for instance, via rising aggregate utilitarian value or empowering stakeholders in innovation processes).
Latulippe *et al*.^57^	**Amartya Sen’s Theoretical Framework of Social Justice** ^58^ provides an interpretative framework for co-design as a democratic process and as a conversion factor. There are three values for the democratic process: intrinsic value, instrumental value (integrated decision-making) and constructive value as a social construct. Conversion factor aids with making sure that the intervention is inclusive.

### Co-creation

Empowerment Theory was used by two articles.^33^^,^^35^ Anselma *et al*.^33^ referenced this theory to Israel *et al*.^34^ for evaluating the influence of the youth-led participatory action research (process evaluation), as linked to children’s empowerment. Specifically, this theory was used for the interview guide, forming provisional codes for data analysis and evaluation. Cueva *et al*.^35^ used the same theory, referencing Perkins and Zimmerman,^36^ to inform a culturally respectful intervention to empower individuals and endorse wellness. This study’s surveys were developed using such theory for intervention evaluation.

One article^37^ used two theories, namely Social Learning Theory and Narrative Theory. It attributes Social Learning Theory to the references of Bandura^38^^,^^39^ as well as Mar and Oatley^40^ and attributes Narrative Learning Theory to Mar and Oatley,^40^ Moyer-Gusé^41^ and Schank and Abelson.^42^ These theories were justified as compatible with the applied photo stories method, adopted as a health literacy intervention and applied over five phases starting with a literature review and stakeholder analysis. Furthermore, this article argued that narrative communication employs story structures that present a familiar point of interaction, ensuring the intervention is easier to process. The photo stories method was underpinned by social learning theory, whilst narrative theory was used in the research planning and production.

One article^43^ utilized ‘Symbolic Interactionism’ as their theoretical framework, referenced to Blumer,^44^ for disentangling the complexities of rehabilitation with multiple stakeholders and regarding how meaning is co-constructed by stakeholders. This framework was used to form an observation guide and the analyses.

### Co-creation and co-design

One article^45^ used ‘Social Effectiveness of Interventions’ theory attributed to Rod *et al*.^46^ to comprehend the social effectiveness of interventions. This theory was employed as an analytical method in co-creation processes and exploring design-based research methodology.

In addition, a specified theoretical ‘realist evaluation’ framework was referenced to Tilley and Pawson^48^^,^^49^ by one article^47^ and was used to account for the strategic changes over time in regard to NCD prevention. It was used to demonstrate the sense-making process that occurs between context, mechanism and outcome patterns of a programme.

### Co-production and co-design

Duke *et al*.^50^ referred to Normalization Process Theory, referenced to May,^51^^,^^52^ which was substantiated by combining participatory learning and action research—believed to retain a positive influence over the design quality of the intervention and implementation, as enabling diverse knowledge and expertise. This theory was used in the pilot implementation phase, as relating to the data collection.

Clarke *et al*.^53^ referred Interactional Ritual Change Theory to Goffman and Collins.^54^ They adopted this theory as pinned to the importance of enabling inclusivity of stakeholders—by highlighting everyday routines/ rituals in relation to more sense of belonging. In addition, the authors suggest that this theory offers a novel perspective regarding situated co-production practices, which was used for the data analysis in exploring how co-production is applied to promote sustainable and inclusive initiatives.

### Co-design

Social Innovation Theory, used by Farmer *et al*.^55^ is referenced to Ayob *et al*.^56^ to consider initiatives co-designed with communities as social innovation, identify helpful initiatives in co-design approaches and aid change in public health. It was also used for exploring how top-down community participation processes permit diverse participants to exchange, for instance, knowledge in new contexts.

Latulippe *et al*.^57^ used Amartya Sen’s theoretical framework of social justice^58^ as informative for co-design in exploring how it enables an inclusive intervention and can lead to the formation of a universally accepted tool (e.g., eHealth tool), for benefitting future users (e.g., caregivers of functionally dependent older people).

## Discussion

### Main finding of this study

Since 2012, 10 articles explicitly used theories for co-creation, co-design and co-production in public health research, comprising six distinct theories and three distinct theoretical frameworks. Empowerment theory was utilized by two different articles, whereas the other theories were used once per article. The ‘how’ and ‘why’ of all theories were outlined, including theory summaries. For instance, most theories used by these articles had informed the methods (focus groups, etc.), with their relevance justified.

### What is already known on this topic?

Recent studies highlighted the popularity of claiming co-creation, co-design and co-production for research, owing to its wide-ranging benefits.^2–4^ Yet, it is evident that the conceptualization behind these co-approaches requires more development.^59^ Specifically, there is a lack of clarity and delineation about how these terms are understood and applied in public health research (and other fields). In turn, this risks knowledge fragmentation and concept stretching. Hence, systematic theory-building into such co-approaches is key.^11–13^

### What this study adds

Our findings can enable theoretically informed public health research applying co-creation, co-design and co-production. For instance, Empowerment Theory can inform how stakeholders can be empowered, as linked to outcomes^33^ and explicit aims.^35^ Some of the same named theories from the public health literature fit with that of other fields that use co-creation, co-design and co-production. For instance, Empowerment Theory was used as a method to understand the concept of customer empowerment and thereby inform about knowledge value co-creation^60^; the Theoretical Framework of Symbolic Interactionism was used to analyse the social interactions between stakeholders involved in a forest management conflict, for instance, as this framework is useful for identifying ways in which role confusion contributes to conflict^61^; and the Normalization Process Theory was used in health services research to support the implementation of complex interventions, by recognizing sense-making, engagement, action and monitoring—and adding to its transparent evaluation.^62^

Most named theories were used once, except for Empowerment Theory used in two co-creation studies, raising the question as to why this theory is not used more often in co-creation research in public health, similar to other fields.^63–66^ This theory may be of closer relevance to co-creation given empowerment is about the importance of agency to influence change. Other theories were only used once which may be because co-creation research in the field of public health is relatively new, albeit evolving. We encourage research to be directed towards all the named theories covered by this review, including whether Empowerment Theory is more relevant than other theories for co-creation. In addition, we recommend future researchers to give clear definitions for creation, co-design and co-production to permit conceptual clarity, and thereby give more strength to the interpretation of its relevant use of theory.

### Limitations of this study

Implicit theories utilized for public health research for co-creation, co-design and co-production may have been missed as this review was solely focused on named theories. The same applies for studies using alternative terms for co-creation, co-design and co-production that incorporated explicit theories in public health research. Furthermore, the articles naming an approach (e.g., co-creation) may not have utilized the same definition of the term as this review. However, our search strategy was optimized through reference list checking and by trialling different search terms for results comparison. The quality assessment tool did not cover ‘low’ score ratings, potentially giving a more positive impression about studies than warranted—yet this rating did not influence our theory analysis.

## Conclusion

Currently, there is a lack of explicitly named theory supporting co-creation, co-design and co-production in public health research. Given a wealth of interest and application geared towards co-creation, co-design and co-production research, more progress needs to be made in relation to the incorporation of named theory/ theoretical frameworks informing these co-approaches. This review helped to identify some usable theories and theoretical frameworks, in addition to delineating how and why the theories were used for such co-approaches. Thus, public health researchers can contemplate the differing applications and justifications for the use of such theories and theoretical frameworks in this way.

### Originality

This article is an original piece of work which has not been published before.

## Data Availability

The protocol of this systematic review was submitted to PROSPERO, under registration ID: CRD42022324074. The dissemination outputs are provided to the Health CASCADE network members as a means of raising awareness and promoting knowledge about the contemporary theories used for co-creation, co-design and co-production in public health.

## Notes

**Katrina Messiha,** MSc (PhD fellow)

**Mai J.M. Chinapaw,** Professor, Dr

**Hans C.F. Ket,** Medical Information Specialist

**Qingfan An,** MSc, (PhD fellow)

**Vinayak Anand-Kumar,** MSc (PhD fellow)

**Giuliana R. Longworth,** MSc (PhD fellow)

**Sebastien Chastin,** Professor, Dr

**Teatske M. Altenburg,** Assistant Professor, Dr

## Appendix 1 Detailed search strategy

**Table:**

PubMed 11 March 2022 (theory: 294, paradigm: 43, framework: 167, concept: 139, principle: 72, model: 163)			
Search	Query	Results	Sample set
#17	**(#4 AND #16) NOT (#5 OR #9 OR #11 OR #13 OR #15)**	**163**	Model
#16	**model*[tiab]**	**3 361 754**	
#15	**(#4 AND #14) NOT (#5 OR #9 OR #11 OR #13)**	**72**	Principle
#14	**princip*[tiab]**	**506 843**	
#13	**(#4 AND #12) NOT (#5 OR #9 OR #11)**	**139**	Concept
#12	**concept*[tiab]**	**536 694**	
#11	**(#10 AND #4) NOT (#5 OR #9)**	**167**	Framework
#10	**framework[tiab] or "frame-work"[tiab]**	**313 340**	
#9	**(#4 AND #8) NOT #5**	**43**	Paradigm
#8	**paradigm*[tiab]**	**158 140**	
#7	**#5 AND #6**	**19**	
#6	**"public health*"[tiab]**	**326 458**	
#5	**#2 AND #4**	**294**	Theory
#4	**"co-creat*"[tiab] OR "cocreat*"[tiab]**	**1692**	
#3	**#1 AND #2**	**979**	
#2	**"theor*"[tiab]**	**742 371**	
#1	**"co-creat*"[tiab] OR "co-design*"[tiab] OR "co-produc*"[tiab] OR "cocreat*"[tiab] OR "codesign*"[tiab] OR "coproduc*"[tiab]**	**9522**	
#5 (294 refs) has relevant items, the other samples do not.			

**Table:**

Sample set co-design/ co-production PubMed 28 Mar 2022 (136)		
Search	Query	Results
#9	**#8 NOT #3**	**136**
#8	**#4 AND #7**	**140**
#7	**"theor*"[ti] OR "theor*"[ot]**	**157 676**
#6	**#5 NOT #3**	**690**
#5	**#2 AND #4**	**709**
#4	**"co-design*"[tiab] OR "co-produc*"[tiab] OR "codesign*"[tiab] OR "coproduc*"[tiab]**	**8084**
#3	**#1 AND #2**	**297**
#2	**"theor*"[tiab]**	**744 810**
#1	**"co-creat*"[tiab] OR "cocreat*"[tiab]**	**1714**
#9 (136 refs) has relevant items.		

**Table:**

***Ebsco/ APA PsycINFO 6 Apr 2022 (1250)***			
**#**	**Query**	**Limiters/ expanders**	**Results**
**S2**	(TI("theor*") OR AB("theor*") OR KW("theor*")) AND (TI("co-creat*" OR "cocreat*" OR "co-design*" OR "co-produc*" OR "codesign*" OR "coproduc*") OR AB("co-creat*" OR "cocreat*" OR "co-design*" OR "co-produc*" OR "codesign*" OR "coproduc*") OR KW("co-creat*" OR "cocreat*" OR "co-design*" OR "co-produc*" OR "codesign*" OR "coproduc*"))	Limiters—publication year: 2012–2022	**1250**
**S1**	(TI("theor*") OR AB("theor*") OR KW("theor*")) AND (TI("co-creat*" OR "cocreat*" OR "co-design*" OR "co-produc*" OR "codesign*" OR "coproduc*") OR AB("co-creat*" OR "cocreat*" OR "co-design*" OR "co-produc*" OR "codesign*" OR "coproduc*") OR KW("co-creat*" OR "cocreat*" OR "co-design*" OR "co-produc*" OR "codesign*" OR "coproduc*"))		**1664**

**Table:**

***Ebsco/ CINAHL 6 Apr 2022 (459)***			
**#**	**Query**	**Limiters/ expanders**	**Results**
**S2**	(TI("theor*") OR AB("theor*") OR KW("theor*")) AND (TI("co-creat*" OR "cocreat*" OR "co-design*" OR "co-produc*" OR "codesign*" OR "coproduc*") OR AB("co-creat*" OR "cocreat*" OR "co-design*" OR "co-produc*" OR "codesign*" OR "coproduc*") OR KW("co-creat*" OR "cocreat*" OR "co-design*" OR "co-produc*" OR "codesign*" OR "coproduc*"))	Limiters—publication year: 2012–2022	**459**
**S1**	(TI("theor*") OR AB("theor*") OR KW("theor*")) AND (TI("co-creat*" OR "cocreat*" OR "co-design*" OR "co-produc*" OR "codesign*" OR "coproduc*") OR AB("co-creat*" OR "cocreat*" OR "co-design*" OR "co-produc*" OR "codesign*" OR "coproduc*") OR KW("co-creat*" OR "cocreat*" OR "co-design*" OR "co-produc*" OR "codesign*" OR "coproduc*"))		**523**

**Table:**

***Elsevier/ Scopus 6 Apr 2022 (2735)***		
History count	Search terms	Results
#7	#5 AND (LIMIT-TO (PUBYEAR, 2018) OR LIMIT-TO (PUBYEAR, 2017) OR LIMIT-TO (PUBYEAR, 2016) OR LIMIT-TO (PUBYEAR, 2015) OR LIMIT-TO (PUBYEAR, 2014) OR LIMIT-TO (PUBYEAR, 2013))	**1204**
#6	#5 AND (LIMIT-TO (PUBYEAR, 2022) OR LIMIT-TO (PUBYEAR, 2021) OR LIMIT-TO (PUBYEAR, 2020) OR LIMIT-TO (PUBYEAR, 2019))	**1531**
#5	#1 AND (LIMIT-TO (SUBJAREA, "SOCI") OR LIMIT-TO (SUBJAREA, "MEDI") OR LIMIT-TO (SUBJAREA, "NURS") OR LIMIT-TO (SUBJAREA, "HEAL"))	**3211**
#1	TITLE-ABS-KEY (("theor*") AND ("co-creat*" OR "cocreat*" OR "co-design*" OR "co-produc*" OR "codesign*" OR "coproduc*"))	**8673**

## Appendix 2 Quality assessment extraction table

**Table:**

Study reference	Is the methodology identified and justified? *(for example, why PAR?)*	Was a theoretical lens/ perspective used to guide the study, with a reference provided?	Is the theory* described? (sufficient for a summary of the theory content to have been given—such as a definition/ reference attributed to the theory*)	Is the theory* easily linked to co-creation, co-design and co-production?	If an explicit theoretical framework is used *(the named framework would need to be expressly stated as theoretical),* are the concepts adequately defined?	Are the relationships among the concepts clearly operationalized for the context under study?	TOTAL # = YES	METHODOLOGICAL QUALITY* OUTCOME
Anselma *et al*. (2020)	Yes	Yes	Yes	Partial	N/A	Yes	4	Strong
Cueva *et al*. (2017)	Partial	Yes	Yes	Yes	Yes	Partial	4	Strong
Koops van’t Jagt *et al*. (2016)	Yes	Yes	Yes	Yes	N/A	Yes	5	Strong
Handberg *et al*. (2019)	Yes	Yes	Yes	Yes	Partial	Yes	5	Strong
Hoeeg *et al*. (2019)	Yes	Yes	Yes	No	N/A	Partial	3	Moderate
Garton *et al*. (2022)	Yes	Yes	Yes	Yes	Yes	Yes	6	Strong
Duke *et al*. (2020)	Partial	Yes	Yes	Yes	Yes	Yes	5	Strong
Clarke *et al*. (2019)	Yes	Yes	Yes	Yes	N/A	Partial	4	Strong
Farmer *et al*. (2018)	Yes	Yes	Yes	Yes	N/A	Partial	4	Strong
Latulippe *et al*. (2020)	Partial	Yes	Yes	Yes	Partial	Partial	3	Moderate
